# Transcriptomic analysis reveals a WNT signaling pathway-based gene signature prognostic for non-small cell carcinoma

**DOI:** 10.18632/aging.103724

**Published:** 2020-10-07

**Authors:** Gang Liu, Wenhui Xie, Mingming Jin, Ping Li, Liu Liu, Lei Liu, Gang Huang

**Affiliations:** 1 Institute of Biological Sciences, Fudan University, Shanghai, P.R. China; 2 Shanghai Key Laboratory of Molecular Imaging, Shanghai University of Medicine and Health Sciences, Shanghai, P.R. China; 3 Department of Nuclear Medicine, Shanghai Chest Hospital, Shanghai Jiaotong University, Shanghai, P.R. China

**Keywords:** NSCLC, prognosis, WNT, model

## Abstract

The value of combining multiple candidate genes into a panel to improve biomarker performance is increasingly emphasized. Genes associated with WNT signaling are widely-reported to provide prognostic signatures in non-small cell carcinoma (NSCLC). Screening of genes involved in this signaling pathway facilitated selection of an optimal candidate biomarker gene combination and development of an NSCLC prognostic model based on expression of these genes. Risk scores derived from the model performed well in predicting survival; in the training dataset, samples achieving a high risk score exhibit a shorter survival interval (median survival time 34.8 months, 95% CI 31.1-41.0) than did samples achieving a low risk score (median survival time 72.0 months, 95% CI 59.3-87.5, p=2e-11), and exhibited higher oncogene and lower tumor suppressor gene expression. Receiver-operator characteristic curves based on three-year survival demonstrate that the model outperformed clinical prognostic indicators. In addition, the model was validated in four independent cohorts, demonstrating robust NSCLC prognostic value. Correlation analyses reveal that the model offers efficacy independent of other clinical indicators. Gene Set Enrichment Analysis (GSEA) reveals that the model reflects variable tissue functional states relevant to NSCLC biology. In summary, the signature model shows potential as a valuable and robust NSCLC prognostic indicator.

## INTRODUCTION

Lung cancer is the most common type of cancer worldwide, exhibiting a five-year survival rate of less than 15% [2]. In China, an estimated 733,300 incident cases and 610,200 deaths occurred in China in 2015 [1]. Non-small cell lung cancer (NSCLC) - a heterogeneous condition [5] consisting mainly of lung adenocarcinoma (LUAD) and lung squamous cell carcinoma (LUSC) subtypes - accounts for the majority (85%) of lung cancer cases [3]. Despite recognition of tobacco smoke as the major causative agent of lung cancer [4], exact pathogenic mechanisms remain unclear. Thus, identification of effective NSCLC prognostic biomarkers is urgently required.

Previously-identified NSCLC prognostic biomarker candidates include various messenger RNAs (mRNAs), microRNAs (miRNAs), long non-coding RNAs (lncRNAs), and certain genes. For example, a study of 154 NSCLC patients reports that miRNA-26b represents both a diagnostic and a prognostic indicator [9]. Similarly, certain lncRNAs are associated with NSCLC tumor size, differentiation, lymph node metastasis, and poor prognosis [10]. Regarding protein-coding genes, tissue MYC Proto-Oncogene (MYC) expression correlates significantly with Programmed Cell Death 1 (PDCD1) expression, and predicts NSCLC patient survival [6]. According to a retrospective study of 218 NSCLC patients, dysregulated Olfactomedin 4 (OLFM4) expression correlates significantly with decreased overall and disease-specific survival (DSS) [7]. Similarly, high-Mobility Group AT hook 2 (HMGA2) correlates with clinical features, lymph node metastasis, and poorer overall survival (OS) [8].

Because genes belonging to a common signaling pathway have complementary functions, integrating information regarding expression of *multiple* genes effectively decreases the impact of NSCLC genetic heterogeneity on prognostication. The Wnt/β-catenin signaling pathway is implicated in regulating NSCLC progression, thus influencing prognosis [11]. Additionally, it has been demonstrated that genes involved in regulating WNT signaling also have NSCLC prognostic value. For example, up-regulated expression of HIF-2α-dependent lncRNA NEAT1 activates WNT signaling, thereby promoting NSCLC progression [12]. Similarly, Forkhead Box Protein P3 (encoded by FOXP3) also activates WNT signaling, facilitating proliferation and metastasis of NSCLC [13]. A similar function has been reported for Telomere-Associated Protein RIF1 (encoded by RIF1) [14].

However, clinical utility of the above biomarker candidates remains limited, owing to NSCLC molecular heterogeneity and relatively small study sample sizes, resulting in relatively low biomarker robustness. It is likely that a multi-gene biomarker panel focused on WNT signaling will outperform any single biomarker in accurately predicting NSCLC prognosis. The present study accessed data from five multi-center clinical cohorts to identify NSCLC prognostic biomarker candidates, and evaluated performance of the resulting multi-gene model.

## RESULTS

### Candidate gene screening and model development

Transcriptomic and clinical data from five independent primary NSCLC cohorts were retrieved from public databases, including The Cancer Genome Atlas (TCGA) and the Gene Expression Omnibus (GEO). Different cohorts employed different gene expression assay platforms. The largest of these cohorts (the training cohort) – deriving from TCGA - was used to identify candidate prognostic biomarker genes and develop a prognostic model based on their expression level.

Briefly, signaling pathway gene member expression was evaluated for significant correlation (p < 0.01) with overall survival (OS). Panels of 2-7 combined candidate genes were used to generate prognostic models, producing risk scores predictively stratifying training cohort samples into high- and low-risk groups (risk score and survival data are detailed in Supplementary Table 1). Model prognostic performance was evaluated by calculating significance (p < 0.05) of survival difference between risk score-predicted high- and low-risk groups, retaining the best-performing prognostic model for survival prediction.

The best-performing panel consisted of seven genes belonging to the WNT signaling pathway: *SFRP1*, *CSNK1E*, *CAMK2A*, *CCND2*, *FOSL1*, *PPP2R1A*, and *PPARD* (Table 1). Expression of these seven genes exhibited differential regulation in cancerous as compared to paired non-cancerous tissue (Supplementary Figure 1). Based on the model (Table 2), risk score was calculated as (0.04289*SFRP1) + (0.08538*CSNK1E) + (0.05043*CAMK2A) + (-0.12109*CCND2) + (0.09305*FOSL1) + (0.23973*PPP2R1A) + (0.04935*PPARD). Positive values indicate that high expression of those genes predict poorer survival, while negative values indicate that high expression of those genes predict improved survival.

**Table 1:** Model gene coefficients as assessed by Cox multivariate regression, where b-value represents coefficient of the model.

**Genes**	**HR**	**95%CI**	**p-value**	**b-value**
PPARD	1.05	0.88~1.26	0.59	0.05
PPP2R1A	1.27	1.02~1.59	0.03	0.24
FOSL1	1.10	1.04~1.16	0.00	0.09
CCND2	0.89	0.83~0.95	0.00	-0.12
CAMK2A	1.05	0.97~1.14	0.21	0.05
CSNK1E	1.09	0.91~1.31	0.36	0.09
SFRP1	1.04	1.00~1.08	0.03	0.04

**Table 2:** Prognostic performance of gene combinations.

**Combination**	**pvalue**
SFRP1,CSNK1E,CAMK2A,CCND2,FOSL1,PPP2R1A,PPARD	1.04E-11
UBE2G1,CDC20,WWP1,TRIM32,HERC4,UBE2C	0.006474
SERPINB5,RRM2,CHEK1,SERPINE1,THBS1	0.007014
MAD2L1,HSP90AA1,CCNA2,BUB1,CDC25C,CCNB2,CCNB1	0.007968
GRIA1,NTSR1,VIPR1	0.00961
CDC20,WWP1,TRIP12	0.00962
AK2,CANT1,PDE6B,GUCY1A3,RRM2,ENTPD3	0.009938

### Performance of the WNT pathway gene prognostic model in the training cohort

**Figure 1:**
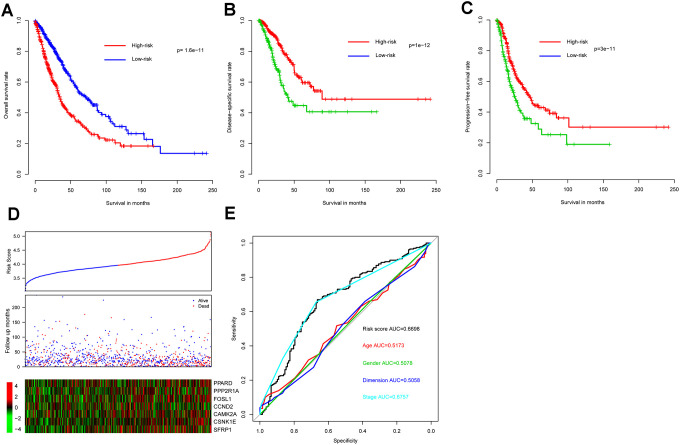
**Model prognostic performance in the training cohort.** Overall (**A**), disease-free (**B**), and progression-free (**C**) survival intervals were compared between risk score-predicted high- and low-risk groups. Detailed survival, risk score, and gene expression pattern data are shown (**D**). The heatmap shows scaled relative gene expression values for each sample. Area under the receiver-operator characteristic (AUROC) curve for clinical indicator- and risk score-based survival prediction is shown (**E**).

As expected, OS interval was significantly lower in the high-risk group (median survival 34.8 months, 95% CI 31.1-41.0) than in the low-risk group (median survival 72.0 months, 95% CI 59.3-87.5) (*p* = 2e-11) (Figure 1A). In addition, consistent with these results, DSS and progression-free survival (PFS) interval differences between high- and low-risk groups indicated that risk score correlated significantly with both (*p* = 1e-12 and p = 3e-11, respectively) (Figure 1B, 1C). Metadata demonstrated that high-risk samples are characterized by earlier incidence, higher oncogene expression, and lower tumor suppressor gene expression (Figure 1D).

Risk score performance was further evaluated - relative to performance of demographic and clinical indicators using receiver-operator characteristic (ROC) curves based on three-year survival. The area under the curve (AUC) for risk score, age, gender, tumor size, and stage was 0.6698, 0.5173, 0.5078, 0.5058, and 0.6757, respectively, indicating that stage and risk score outperformed the other indicators in discriminating prognosis. Since *p*-values for genes *PPARD*, *CAMK2A*, and *CSNK1E* exceed 0.05 (Table 1) when applying Cox multivariate regression, a model incorporating only the remaining four (significantly associated) genes was also constructed. However, since this resulted in a significant survival difference between high- and low-risk groups in only one out of five cohorts (Supplementary Figure 2) the other three genes do appear to contribute to NSCLC prognosis and the full seven-gene model was retained.

Within the training cohort, 28 patients exhibited distant metastasis, a clinically important indicator of the extent of NSCLC progression. Although risk scores of the metastasis-positive group trended higher than those of the metastasis-negative group, differences were not statistically significant (*p* = 0.1, data not shown).

Collectively, these results indicate that the WNT signaling gene expression-based model is effective in predicting NSCLC survival.

### Validation of risk score performance using independent cohorts

**Figure 2:**
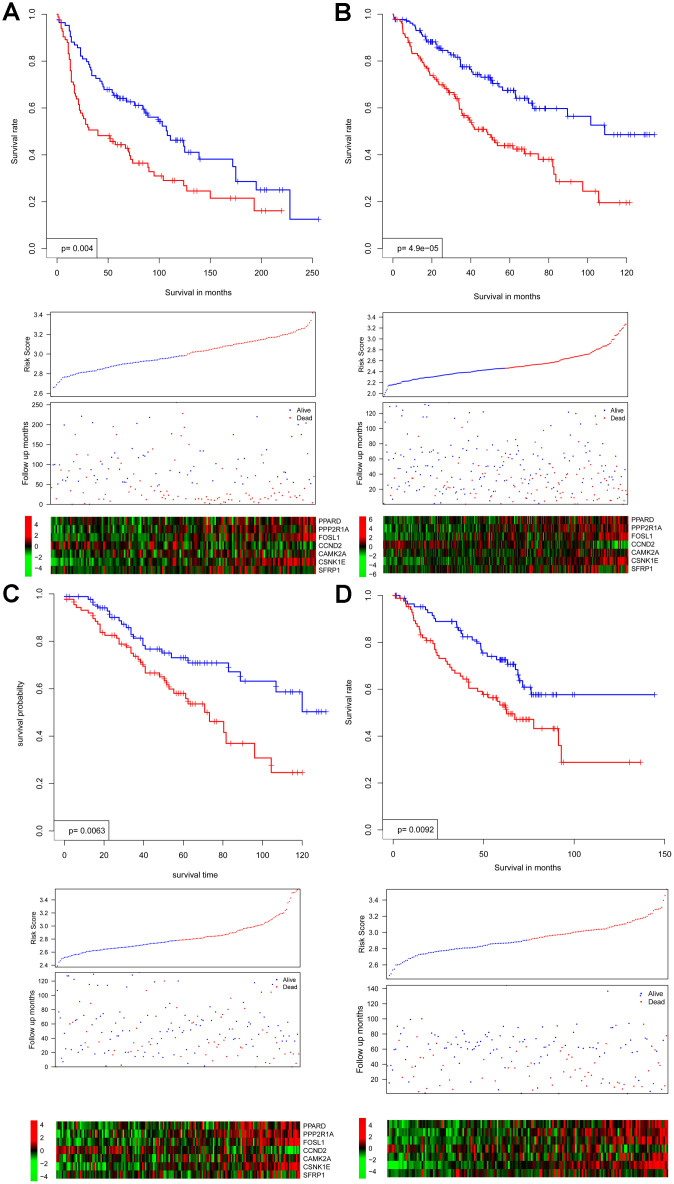
Survival interval between risk score-predicted high- and low-risk groups (upper panel) was compared in independent cohorts GSE30219 (**A**), GSE4127 (**B**), GSE42127 (**C**), and GSE50081 (**D**). Detailed survival, risk score, and gene expression pattern data are shown (lower panel).

In all independent cohorts, survival intervals are significantly lower in the high-risk group (*p* = 0.004, 4.9e-5, 0.0063, and 0.0092, respectively) (Figure 2A–2D). Consistent with this observation, gene expression and survival patterns of the independent cohorts resemble those of the training cohort.

Collectively, these results indicate that the WNT signaling gene expression-based model is robust, performing reproducibly across independent NSCLC cohorts and gene expression platforms.

### Risk score characterization

**Figure 3:**
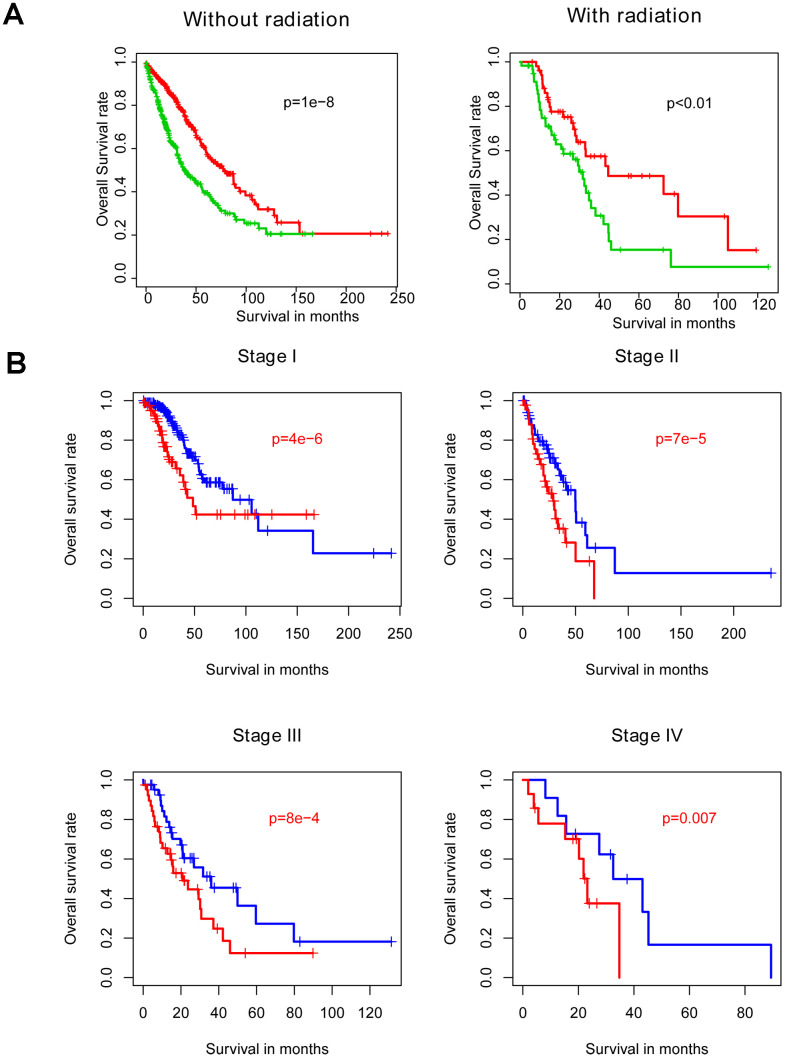
****Within-subgroup prognostic value of the risk score, for radiotherapy-treated and –untreated (**A**) samples, as well as for samples stratified by stage (**B**).

Firstly, risk score was analyzed for correlation with existing clinical indicators (variables), and risk score is not significantly associated with age, tumor size, or pathological stage (Figure 3A). Furthermore, Cox multivariate regression was implemented using risk score as well as clinical indicators (Figure 3B). Results suggest that risk score has value independent from clinical indicators in predicting NSCLC clinical outcome.

### Model bias estimation within subgroups

**Figure 4:**
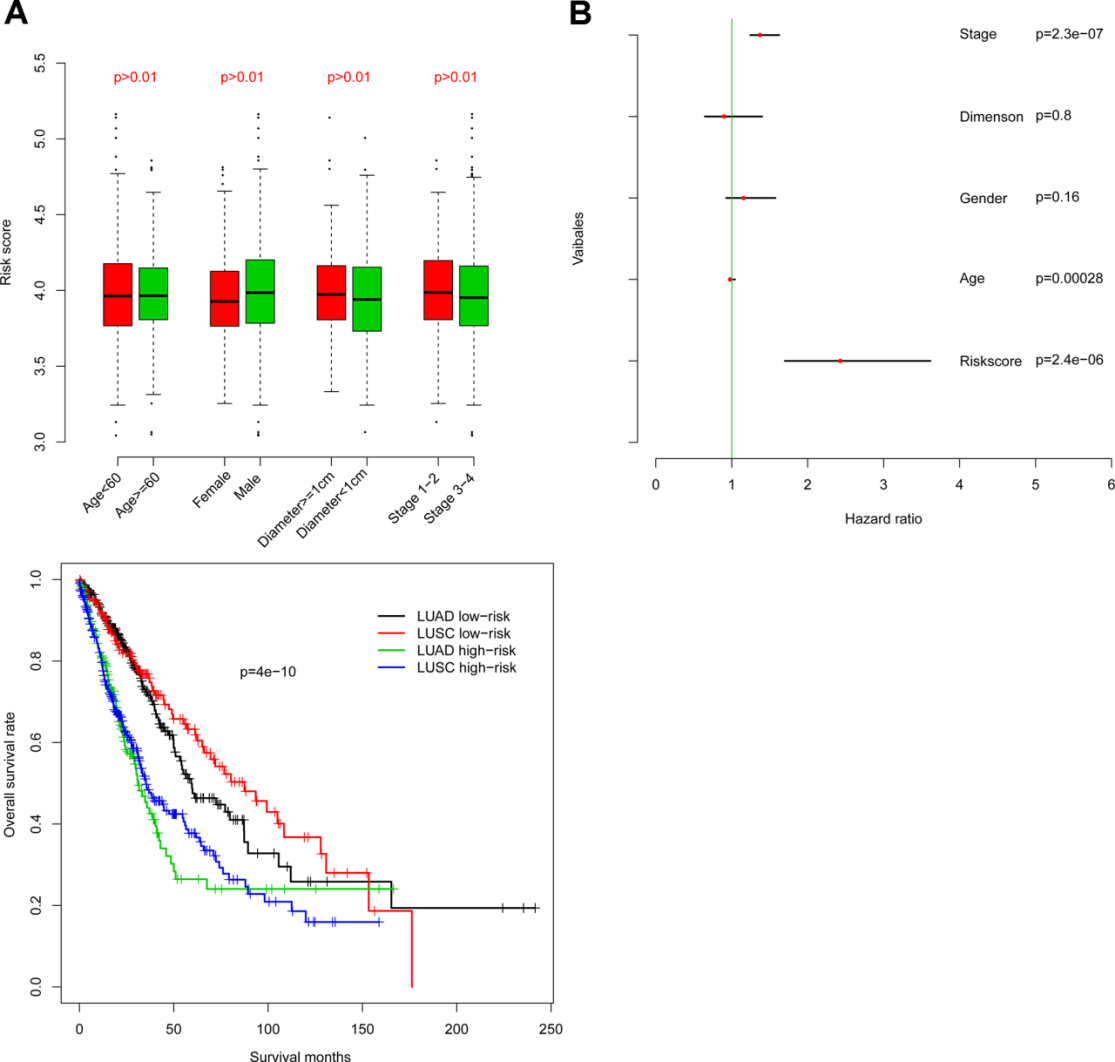
**Comparison of the prognostic value of risk score relative to other clinical indicators.** Risk score is independent of other clinical indicators (**A**) and is significantly associated with OS as assessed by Cox multivariate regression (**B**).

Radiotherapy is one the most important adjuvant therapies offered to NSCLC patients. Thus, samples were divided into radiotherapy-treated and –untreated subgroups for within-subgroup evaluation of risk score performance. Consistent with abovementioned results, both high-risk groups exhibited longer survival intervals in the training cohort, relative to the low-risk groups (Figure 4A). Similarly, analyzing samples subdivided according to stage (Figure 4B) or LUAD versus LUSC subtype (Figure 4C) demonstrated no model bias in predicting prognosis.

Results indicate that risk score performs favorably and is independent of existing clinical indicators, at least one treatment modality, and clinical as well as pathological disease subtypes.

### Risk score reflects biological significance of gene expression patterns

**Figure 5:**
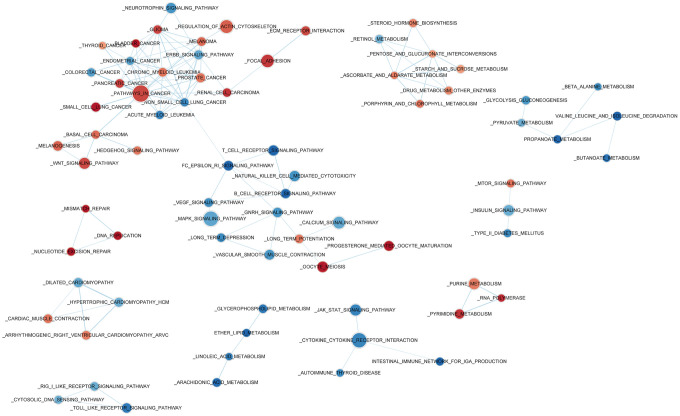
**Enriched biological pathways associated with the risk score.**

Gene Set Enrichment Analysis (GSEA) was used to investigate high- and low-risk group transcriptomic data biological meaning, and results were visualized using the Cytoscape plugin EnrichmentMap. Enriched biological cassettes include up-regulation of cancer-promoting pathways, and down-regulation of cancer- suppressing pathways (Figure 5). Results indicate that the gene expression-based risk score reflects the biological status of pathways underlying NSCLC.

## DISCUSSION

Relevance of WNT signaling pathway functions has been widely emphasized for various cancers [11, 15, 16], including NSCLC. However, due to genetic and environmental heterogeneity [17–19] at all biological levels, even within the same tumor, it is anticipated that integrating information from multiple genes belonging to the WNT signaling pathway may significantly improve prognostication. The present study identifies an optimized panel of candidate biomarkers consisting of genes belonging to the WNT signaling pathway, and develops and evaluates a prognostic model based on expression of these genes.

The model incorporates seven genes: *SFRP1*, *CSNK1E*, *CAMK2A*, *CCND2*, *FOSL1*, *PPP2R1A*, and *PPARD*. Many of these likely play roles in cancer progression. For example, *SFRP1* is known to inhibit Epithelial to Mesenchymal Transition (EMT) [20] in NSCLC cell lines, and has previously been suggested as a potential biomarker for NSCLC [21]. The prognostic role of *CCND2* has been frequently reported in many cancer types [22], though effects are context specific. For example, it acts as a tumor suppressor gene in NSCLC, while acting as an oncogene in gastric, bladder, and breast cancers. Functions of the remaining genes - including *CSNK1E* [23], *FOSL1* [24], *PPP2R1A* [25], and *PPARD* [26] – are widely reported as being relevant to cancer (if not specifically NSCLC) biology.

## MATERIALS AND METHODS

### Sample enrollment and data processing

Primary NSCLC transcriptomic and clinical metadata from five independent cohorts - deriving from multiple centers employing different gene expression assay platforms - were retrieved from the GEO (Gene Expression Ominous) and TCGA (The Cancer Genome Atlas) public databases, requiring no additional informed consent or ethical approval.

Raw transcript counts downloaded from GEO were log_2_ transformed and normalized as described below. Detailed assay platform, sample number, tumor type, country, and normalization method data are shown in Supplementary Table 6. Raw transcript counts for the TCGA cohort were downloaded using the University of California Santa Cruz (UCSC) Xena tool (https://xena.ucsc.edu/) and log_2_ transformed after 1% minimum value imputation. Only samples associated with complete survival information were included, with such clinical metadata consisting of three types of carefully curated survival endpoints: OS (overall survival), DSS (disease-specific survival, PFS (progression-free survival).

### Candidate gene screening and prognostic model development and evaluation

Due to its large sample size, the TCGA cohort was used as the training cohort to identify candidate prognostic biomarker genes.

Over 170 human signaling pathways were downloaded from the Kyoto Encyclopedia of Genes and Genomes (KEGG) database (https://www.kegg.jp/) in order to generate lists of each pathway’s member genes. Correlation between OS and gene expression was evaluated using Cox univariate regression analyses (as described below). Genes with a *p*-value of less than 0.01 were deemed significantly associated with OS and were retained as candidate prognostic biomarkers for downstream analysis.

For each pathway, a matrix of all possible remaining member gene combinations was generated. Although some pathways retained over 40 member genes significantly associated with OS, subsequent combinatorial evaluation of panel performance was restricted to 2-7 genes per combination, in order to remain within available computational resource constraints, as well as to facilitate eventual clinical utility. Multivariate Cox regression analysis was implemented for each panel, and risk score models were developed based on linear formula coefficients in combination with gene expression values. Risk score provided by a model was defined as $\sum_{i=1}^{n}b_{i}*x_{i},$ where b_i_ is the coefficient of gene x and x_i_ is the expression value of that gene. In each case, training cohort samples were divided into high- and low-risk groups using median risk score as the threshold value. The performance of each model was then evaluated by determining significance of survival difference (incorporating all three survival metrics) between risk score-predicted high- and low-risk groups. The best-performing model was retained.

Risk score performance was further evaluated relative to performance of demographic and clinical indicators (age, gender, primary tumor size represented by tumor diameter, and pathological stage) using receiving operating characteristic ROC curves based on three-year survival. Finally, risk score performance was evaluated with respect to training cohort subgroups. The training cohort was divided into metastasis-negative and -positive subgroups, and between-group risk score predictive performance was compared.

Since the predictive model was derived *from* the training cohort, favorable risk score performance is expected in this cohort. Thus, model robustness was validated using four independent GEO cohorts: GSE30219, GSE4127, GSE42127 and GSE50081. As described for the training cohort, gene expression values and coefficients were adapted to generate a risk score for each sample (Supplementary Tables 2–5), median risk score was used as the threshold value discriminating high- and low-risk groups, and model performance was evaluated by significance of survival difference between groups. Additionally, risk score was analyzed for correlation with existing clinical indicators (variables), first binarizing each variable using subjective cut-off values. Age was divided into younger (< 60 years) and older (≥ 60 years) groups, primary tumor size was divided into small (< 1 cm diameter) and large (≥ 1 cm diameter) groups, and pathological stage was divided into early (stage 1-2) and late (stage 3-4) groups. Finally, risk score performance was evaluated with respect to clinically-relevant independent cohort subgroups, as described for training cohort metastasis-positive versus –negative groups. Here, subgroups included samples deriving from radiotherapy-treated and –untreated patients, samples stratified by stage, and samples stratified by LUAD versus LUSC subtype.

Lastly, Gene Set Enrichment Analysis (GSEA) was used to investigate high- and low-risk group transcriptomic data biological meaning, and results were visualized using the Cytoscape plugin EnrichmentMap.

### Analytical software and statistics

All analyses were performed using the R Project for Statistical Computing (http://www.r-project.org). R package “affy” was used for raw data processing and normalization. R function combn() was used to generate matrices of all possible combinations of genes within a pathway. R packages “survival” and “pROC” [27] were used for survival and ROC analyses, respectively. Heatmap was generated using R package“pheatmap”. Survival differences between risk score-predicted high- and low-risk groups were assessed using Student’s t-test, with a significance threshold of *p* < 0.05. Gene Set Enrichment Analyses was performed with GSEA java software [28], and visualized with Cytoscape [29] plugin EnrichmentMap [30].

## Supplementary Material

Supplementary Figures

Supplementary Table 1

Supplementary Table 2

Supplementary Table 3

Supplementary Table 4

Supplementary Table 5

Supplementary Table 6

## References

[1] Chen W, Zheng R, Baade PD, Zhang S, Zeng H, Bray F, Jemal A, Yu XQ, He J. Cancer statistics in China, 2015. CA Cancer J Clin. 2016; 66:115–32. 10.3322/caac.21338 26808342

[2] Field EA, Field JK. Lung cancer: a potential role for dentists. Br Dent J. 2020; 228:413–14. 10.1038/s41415-020-1418-9 32221439

[3] Molina JR, Yang P, Cassivi SD, Schild SE, Adjei AA. Non-small cell lung cancer: epidemiology, risk factors, treatment, and survivorship. Mayo Clin Proc. 2008; 83:584–94. 10.4065/83.5.584 18452692 PMC2718421

[4] Alberg AJ, Brock MV, Ford JG, Samet JM, Spivack SD. Epidemiology of lung cancer: Diagnosis and management of lung cancer, 3rd ed: American College of Chest Physicians evidence-based clinical practice guidelines. Chest. 2013; 143:e1S–e29S. 10.1378/chest.12-2345 23649439 PMC4694610

[5] Chen Z, Fillmore CM, Hammerman PS, Kim CF, Wong KK. Non-small-cell lung cancers: a heterogeneous set of diseases. Nat Rev Cancer. 2014; 14:535–46. 10.1038/nrc3775 25056707 PMC5712844

[6] Kim EY, Kim A, Kim SK, Chang YS. MYC expression correlates with PD-L1 expression in non-small cell lung cancer. Lung Cancer. 2017; 110:63–67. 10.1016/j.lungcan.2017.06.006 28676221

[7] Su W, Luo L, Wu F, Lai Z, Li X, Xie Z, Tang Z, Yang Z, Liang R. Low expression of olfactomedin 4 correlates with poor prognosis in smoking patients with non-small cell lung cancer. Hum Pathol. 2015; 46:732–38. 10.1016/j.humpath.2015.01.013 25771901

[8] Guo X, Shi J, Wen Y, Li M, Li Q, Li X, Li J. Increased high-mobility group A2 correlates with lymph node metastasis and prognosis of non-small cell lung cancer. Cancer Biomark. 2018; 21:547–55. 10.3233/CBM-170401 29278873 PMC13078311

[9] Jiang LP, Zhu ZT, He CY. Expression of miRNA-26b in the diagnosis and prognosis of patients with non-small-cell lung cancer. Future Oncol. 2016; 12:1105–15. 10.2217/fon.16.21 27033050

[10] Shen QM, Wang HY, Xu S. LncRNA GHET1 predicts a poor prognosis of the patients with non-small cell lung cancer. Eur Rev Med Pharmacol Sci. 2018; 22:2328–33. 10.26355/eurrev_201804_14823 29762836

[11] Stewart DJ. Wnt signaling pathway in non-small cell lung cancer. J Natl Cancer Inst. 2014; 106:djt356. 10.1093/jnci/djt356 24309006

[12] Kong X, Zhao Y, Li X, Tao Z, Hou M, Ma H. Overexpression of HIF-2α-dependent NEAT1 promotes the progression of non-small cell lung cancer through miR-101-3p/SOX9/wnt/β-catenin signal pathway. Cell Physiol Biochem. 2019; 52:368–81. 10.33594/000000026 30845377

[13] Yang S, Liu Y, Li MY, Ng CS, Yang SL, Wang S, Zou C, Dong Y, Du J, Long X, Liu LZ, Wan IY, Mok T, et al. FOXP3 promotes tumor growth and metastasis by activating Wnt/β-catenin signaling pathway and EMT in non-small cell lung cancer. Mol Cancer. 2017; 16:124. 10.1186/s12943-017-0700-1 28716029 PMC5514503

[14] Mei Y, Liu YB, Cao S, Tian ZW, Zhou HH. RIF1 promotes tumor growth and cancer stem cell-like traits in NSCLC by protein phosphatase 1-mediated activation of Wnt/β-catenin signaling. Cell Death Dis. 2018; 9:942. 10.1038/s41419-018-0972-4 30237512 PMC6148239

[15] Zhan T, Rindtorff N, Boutros M. Wnt signaling in cancer. Oncogene. 2017; 36:1461–73. 10.1038/onc.2016.304 27617575 PMC5357762

[16] Krishnamurthy N, Kurzrock R. Targeting the Wnt/beta-catenin pathway in cancer: update on effectors and inhibitors. Cancer Treat Rev. 2018; 62:50–60. 10.1016/j.ctrv.2017.11.002 29169144 PMC5745276

[17] Jamal-Hanjani M, Wilson GA, McGranahan N, Birkbak NJ, Watkins TB, Veeriah S, Shafi S, Johnson DH, Mitter R, Rosenthal R, Salm M, Horswell S, Escudero M, et al, and TRACERx Consortium. Tracking the evolution of non-small-cell lung cancer. N Engl J Med. 2017; 376:2109–21. 10.1056/NEJMoa1616288 28445112

[18] Hensley CT, Faubert B, Yuan Q, Lev-Cohain N, Jin E, Kim J, Jiang L, Ko B, Skelton R, Loudat L, Wodzak M, Klimko C, McMillan E, et al. Metabolic heterogeneity in human lung tumors. Cell. 2016; 164:681–94. 10.1016/j.cell.2015.12.034 26853473 PMC4752889

[19] Zhang Y, Chang L, Yang Y, Fang W, Guan Y, Wu A, Hong S, Zhou H, Chen G, Chen X, Zhao S, Zheng Q, Pan H, et al. Intratumor heterogeneity comparison among different subtypes of non-small-cell lung cancer through multi-region tissue and matched ctDNA sequencing. Mol Cancer. 2019; 18:7. 10.1186/s12943-019-0939-9 30626401 PMC6325778

[20] Ren J, Wang R, Huang G, Song H, Chen Y, Chen L. sFRP1 inhibits epithelial-mesenchymal transition in A549 human lung adenocarcinoma cell line. Cancer Biother Radiopharm. 2013; 28:565–71. 10.1089/cbr.2012.1453 23802127 PMC3741431

[21] Taguchi YH, Iwadate M, Umeyama H. SFRP1 is a possible candidate for epigenetic therapy in non-small cell lung cancer. BMC Med Genomics. 2016 (Suppl 1); 9:28. 10.1186/s12920-016-0196-3 27534621 PMC4989892

[22] Ding ZY, Li R, Zhang QJ, Wang Y, Jiang Y, Meng QY, Xi QL, Wu GH. Prognostic role of cyclin D2/D3 in multiple human Malignant neoplasms: a systematic review and meta-analysis. Cancer Med. 2019; 8:2717–29. 10.1002/cam4.2152 30950241 PMC6558476

[23] Tiong KL, Chang KC, Yeh KT, Liu TY, Wu JH, Hsieh PH, Lin SH, Lai WY, Hsu YC, Chen JY, Chang JG, Shieh GS. CSNK1E/CTNNB1 are synthetic lethal to TP53 in colorectal cancer and are markers for prognosis. Neoplasia. 2014; 16:441–50. 10.1016/j.neo.2014.04.007 24947187 PMC4198690

[24] Vallejo A, Perurena N, Guruceaga E, Mazur PK, Martinez-Canarias S, Zandueta C, Valencia K, Arricibita A, Gwinn D, Sayles LC, Chuang CH, Guembe L, Bailey P, et al. An integrative approach unveils FOSL1 as an oncogene vulnerability in KRAS-driven lung and pancreatic cancer. Nat Commun. 2017; 8:14294. 10.1038/ncomms14294 28220783 PMC5321758

[25] Haines K, Huang GS. Precision therapy for aggressive endometrial cancer by reactivation of protein phosphatase 2A. Cancer Res. 2019; 79:4009–10. 10.1158/0008-5472.CAN-19-1938 31416848

[26] Liu Y, Deguchi Y, Tian R, Wei D, Wu L, Chen W, Xu W, Xu M, Liu F, Gao S, Jaoude JC, Chrieki SP, Moussalli MJ, et al. Pleiotropic effects of PPARD accelerate colorectal tumorigenesis, progression, and invasion. Cancer Res. 2019; 79:954–69. 10.1158/0008-5472.CAN-18-1790 30679176 PMC6397663

[27] Robin X, Turck N, Hainard A, Tiberti N, Lisacek F, Sanchez JC, Müller M. pROC: an open-source package for R and S+ to analyze and compare ROC curves. BMC Bioinformatics. 2011; 12:77. 10.1186/1471-2105-12-77 21414208 PMC3068975

[28] Subramanian A, Tamayo P, Mootha VK, Mukherjee S, Ebert BL, Gillette MA, Paulovich A, Pomeroy SL, Golub TR, Lander ES, Mesirov JP. Gene set enrichment analysis: a knowledge-based approach for interpreting genome-wide expression profiles. Proc Natl Acad Sci USA. 2005; 102:15545–50. 10.1073/pnas.0506580102 16199517 PMC1239896

[29] Shannon P, Markiel A, Ozier O, Baliga NS, Wang JT, Ramage D, Amin N, Schwikowski B, Ideker T. Cytoscape: a software environment for integrated models of biomolecular interaction networks. Genome Res. 2003; 13:2498–504. 10.1101/gr.1239303 14597658 PMC403769

[30] Reimand J, Isserlin R, Voisin V, Kucera M, Tannus-Lopes C, Rostamianfar A, Wadi L, Meyer M, Wong J, Xu C, Merico D, Bader GD. Pathway enrichment analysis and visualization of omics data using g:Profiler, GSEA, cytoscape and EnrichmentMap. Nat Protoc. 2019; 14:482–517. 10.1038/s41596-018-0103-9 30664679 PMC6607905

