# Supplementary material for: Transcriptomic analysis reveals a WNT signaling pathway-based gene signature prognostic for non-small cell carcinoma
Source: Aging (Albany NY). 2020 Oct 7;12(19):19159–72. doi: 10.18632/aging.103724 (PMC7732286; doi:10.18632/aging.103724)
Supplement: Supplementary Figures [file aging-12-103724-s001..pdf]

SUPPLEMENTARY FIGURES

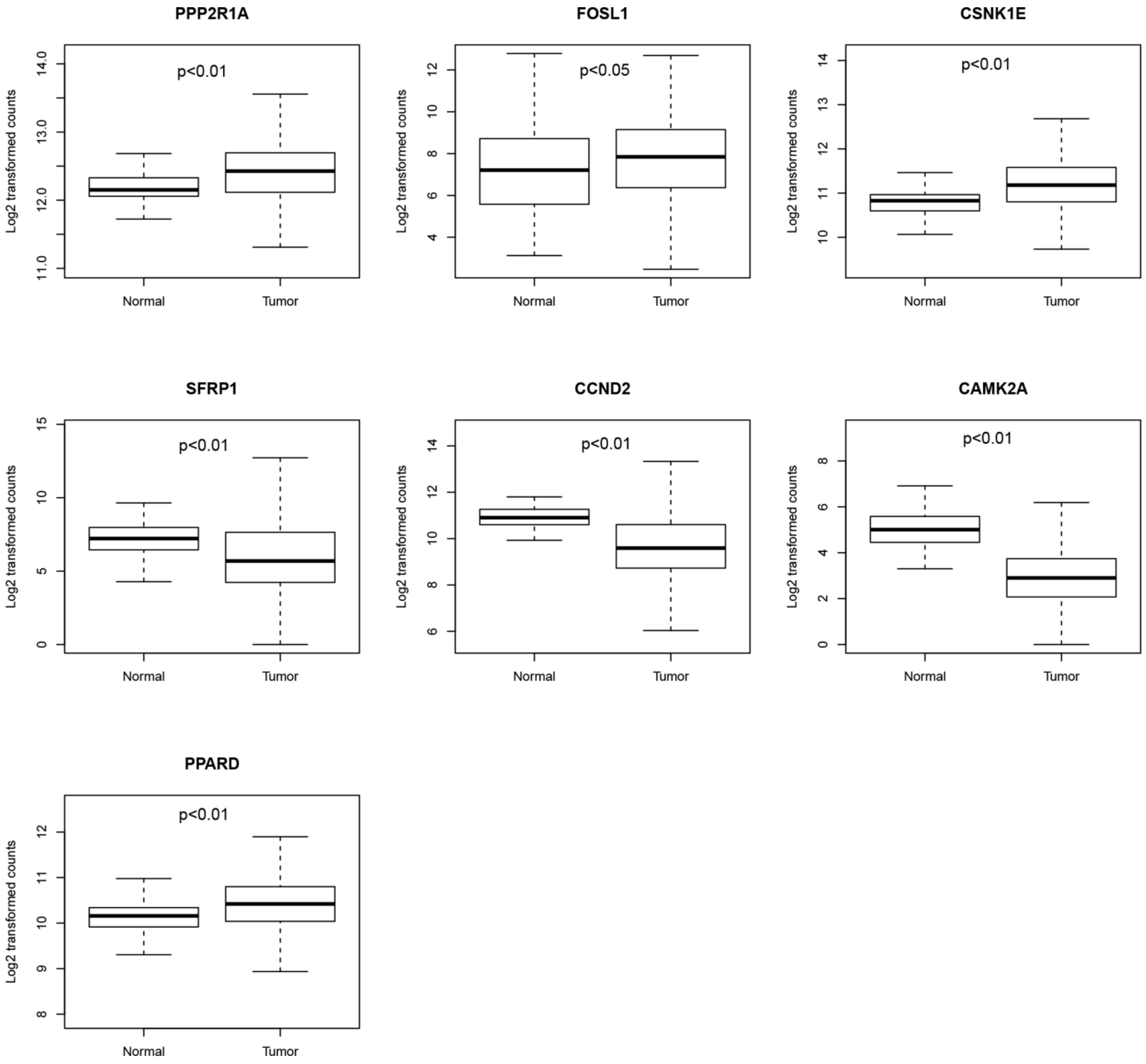

Supplementary Figure 1. Gene expression values of candidate genes in tumor and normal tissues.

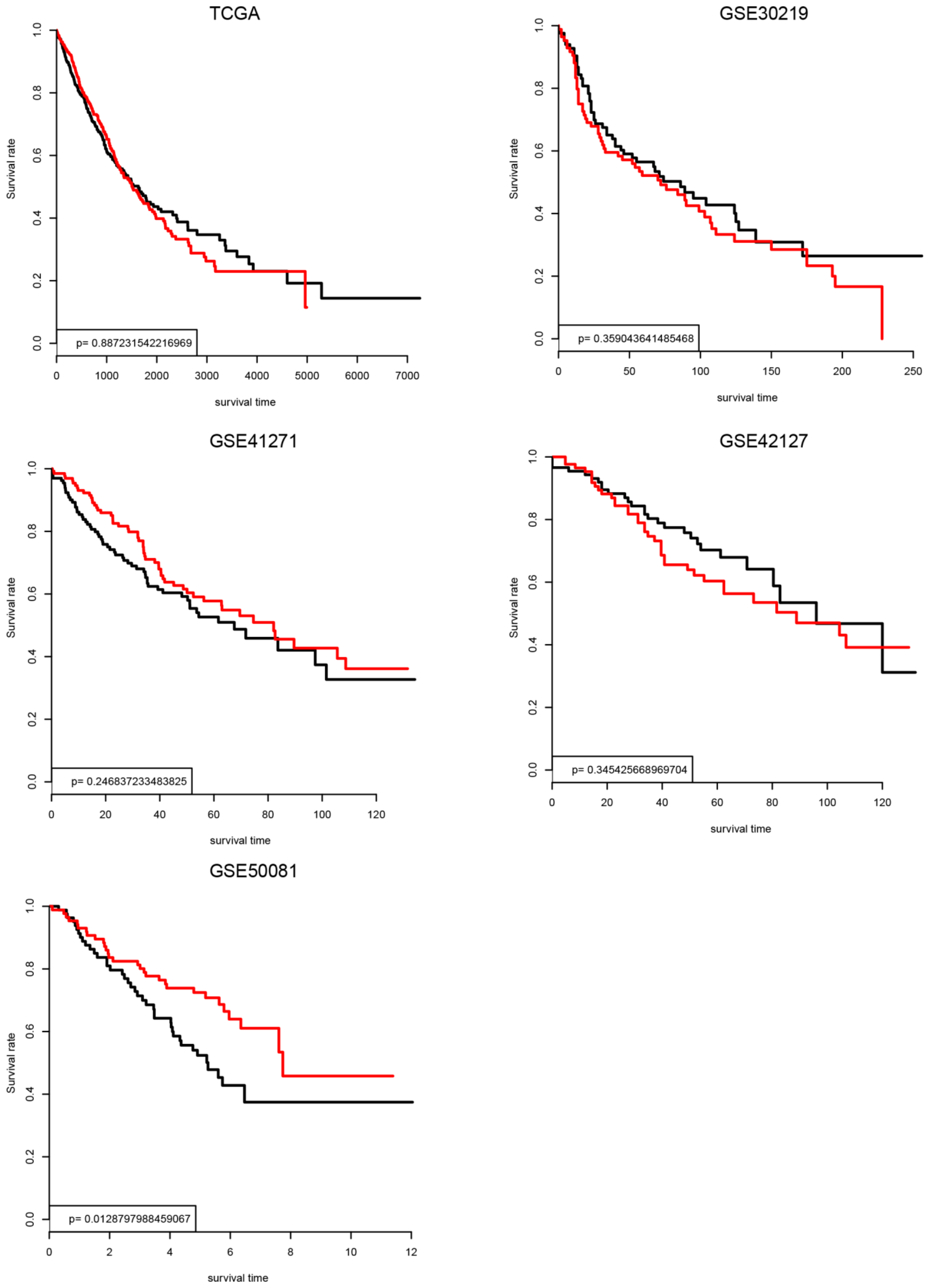

Supplementary Figure 2. Model performance of a four gene model.
