# Supplementary material for: Transcriptomic analysis reveals a WNT signaling pathway-based gene signature prognostic for non-small cell carcinoma
Source: Aging (Albany NY). 2020 Oct 7;12(19):19159–72. doi: 10.18632/aging.103724 (PMC7732286; doi:10.18632/aging.103724)
Supplement: Supplementary Table 6 [file aging-12-103724-s007..pdf]

SUPPLEMENTARY TABLE

Supplementary Table 6. Detailed information for datasets, platform and processing steps.

Name	Platform	SampleNumber	Country	DiseaseType	Normalization
TCGA	IlluminaHiSeq	987	Mostly US	LUSC+LUAD	Log2Counts
GSE30219	AffyU133P2	167	France	LUSC+LUAD+LULN	RMA
GSE41271	IlluminaHumanWG-6v3	264	US	LUSC+LUAD	Quantile
GSE42127	IlluminaHumanWG-6 v3.0	176	US	LUSC+LUAD	Quantile
GSE50081	AffyU133P2	172	Canada	LUSC+LUAD	RMA
